# Can *Macrolophus pygmaeus* (Hemiptera: Miridae) Mitigate the Damage Caused to Plants by *Bemisia tabaci* (Hemiptera: Aleyrodidae)?

**DOI:** 10.3390/insects14020164

**Published:** 2023-02-08

**Authors:** Alessia Farina, Giuseppe Eros Massimino Cocuzza, Pompeo Suma, Carmelo Rapisarda

**Affiliations:** Department of Agriculture, Food and Environment (Di3A), Applied Entomology Section, University of Catania, 95123 Catania, Italy

**Keywords:** whitefly, predator, zoophytophagy, trophic interactions, plant morphology, plant physiology

## Abstract

**Simple Summary:**

The whitefly *Bemisia tabaci* is an invasive pest that causes extensive damage to many vegetable crops and ornamental plants. To control this pest, the release of natural enemies has become increasingly important as an ecologically safe and effective method of biological control. Some species in the family Miridae are effective at controlling whitefly populations, but because they feed on both insect prey and plant tissue, their overall effect on plant performance is not well understood. In this study, the impact of the mirid predator *Macrolophus pygmaeus* on the morphological and physiological traits of *Solanum melongena* in the presence of *Bemisia tabaci* was evaluated. Overall, the results show how the presence of this natural enemy mitigates the damage caused by whitefly infestations. They also help to clarify the multitrophic relationships between plant, pest, and natural enemy, enabling the prediction of plant development in the presence of both pest and predator.

**Abstract:**

Nowadays, in protected vegetable crops, pest management based mainly on biological control represents the most sustainable alternative to pesticide use. The cotton whitefly, *Bemisia tabaci*, is one of the key pests that negatively impact the yield and quality of such crops in many agricultural systems. The predatory bug *Macrolophus pygmaeus* is one of the main natural enemies of the whitefly and is widely used for its control. However, the mirid can sometimes behave as a pest itself, causing damage to crops. In this study, we investigated the impact of *M. pygmaeus* as a plant feeder, by analyzing the combined impact of the whitefly pest and the predator bug on the morphology and physiology of potted eggplants under laboratory conditions. Our results showed no statistical differences between the heights of plants infested by the whitefly or by both insects compared with noninfested control plants. However, indirect chlorophyll content, photosynthetic performance, leaf area, and shoot dry weight were all greatly reduced in plants infested only by *B. tabaci*, compared with those infested by both pest and predator or with noninfested control plants. Contrarily, root area and dry weight values were more reduced in plants exposed to both of the insect species, compared with those infested only by the whitefly or compared with noninfested control plants, where the latter showed the highest values. These results show how the predator can significantly reduce the negative effects of *B. tabaci* infestation, limiting the damage it causes to host plants, though the effect of the mirid bug on the underground parts of the eggplant remains unclear. This information might be useful for a better understanding of the role that *M. pygmaeus* plays in plant growth, as well as for the development of management strategies to successfully control infestations by *B. tabaci* in cropping environments.

## 1. Introduction

Multitrophic interactions, understood as relationships between organisms across different trophic levels of a food web [1,2], are gaining growing interest in ecological studies. Especially in the agricultural sector, which is increasingly oriented to achieve a reduction in chemical inputs, complex interactions that involve the binding of different organisms, living both above and below the ground, with the cultivated plants are now recognized [3,4,5]. Improving our understanding of these relationships may help in planning a rational management of the populations involved, and it may lead to a reduction in pest infestations and their negative effects [6,7,8].

*Bemisia tabaci* (Gennadius) is a notable insect pest that affects vegetable crops and many ornamental plants. It causes damage directly by the piercing of leaves, the suction of sap, and the production of honeydew on which sooty molds develop [9] and also indirectly through its ability to transmit phytopathogenic viruses to numerous host plants [10]. Over 100 virus species belonging to the Begomovirus, Carlavirus, Crinivirus, Ipomovirus, and Torradovirus groups are presently known to be transmitted by *B. tabaci* [11,12], causing worldwide economic damage, the value of which is difficult to estimate. For a long time, *B. tabaci* was considered as a single species, but variability among its populations has led scholars to consider *B. tabaci* as a complex of more than 40 species that are morphologically indistinguishable but that differ in their biology (host range, development performance, suitable environmental conditions, virus transmission capability, etc.) and geographic distribution [13,14,15,16].

Numerous studies have examined the interactions of *B. tabaci* with other components of its food web and how these influence the population levels of the insect, as well as the negative impact of the insect on the host plants. For instance, regarding the interactions of *B. tabaci* with other pests, Tan [17] showed how the infestation of tomato plants by the green peach aphid *Myzus persicae* (Sulzer) had a negative impact on *B. tabaci* development, indicating that the latter clearly prefers to settle on plant leaves not infested by the aphid. A deeper interaction between these two hemipteran species has also been demonstrated by the effect of preinfesting tomato plants with *M. persicae* on the feeding dynamics of *B. tabaci* and on the acquisition and transmission mechanisms of TYLCV (tomato yellow leaf curl virus) by this vector—showing a clear and significant influence of aphid preinfestation on the tomato–whitefly–TYLCV system [18].

At a higher trophic level, whitefly feeding induces plant defense responses, which affect more-complex interactions with natural enemies. Thus, in the whiteflies–aphids system on tomato, a preinfestation with *B. tabaci* MEAM1 impacts the predation ability of the ladybird *Propylea japonica* (Thunberg) on *M. persicae* [19]. At the top of the feeding pyramid, interactions between whiteflies and natural enemies can lead to cases of intraguild predation, which can lower their effectiveness. For instance, the overall predation on the whitefly was reduced when the two mirid bugs, *Macrolophus pygmaeus* (Rambur) and *Nesidiocoris tenuis* Reuter, occurred together on the same plant [20]. On the other hand, when feeding on plants, the two zoophytophagous mirids stimulate plants to release volatile organic compounds (VOCs), which repel pests, such as *B. tabaci* and *Frankliniella occidentalis* Pergande, but attract whitefly parasitoids, such as *Encarsia formosa* Gahan [21].

The complex interactions between plants and phytophagous insects, from which physiological, morphological, or behavioral plasticity derives in both hosts and herbivores, have been the focus of numerous studies [22]. For *B. tabaci*, interactions with plants are extremely important, and they are based on the attractiveness of plants, which is communicated to whiteflies by both visual and (to a lesser extent) biochemical cues. The nutrient composition of host plants therefore impacts whitefly performance, such that nutrient changes or stresses in plants affect nutrition in whiteflies [23]. In turn, whitefly feeding alters the physiology and morphology of plants, causing changes in physiological (e.g., gas exchange, chlorophyll fluorescence, indirect chlorophyll content), biochemical (e.g., enzymes, phenols, flavonoids), or morphological (e.g., plant height, leaf area, shoot dry weight, root dry weight) parameters [24,25,26,27,28,29,30,31,32]. Most of these phenomena remain poorly understood and therefore need to be more deeply investigated in order to improve sustainable whitefly management.

In one previous work, the impact of *B. tabaci* MED on eggplants and tomatoes was investigated. This study considered the principal morphological and physiological traits (e.g., plant height, dry plant biomass, chlorophyll content, etc.) [33] and found that eggplant and tomato plants infested by the whitefly showed strong and significant reductions in height, shoot dry weight, leaf area, and indirect chlorophyll content, though with different levels of intensity among the two plant species. Starting from the above results, and in order to widen our knowledge of multitrophic interaction mechanisms related to the impact of whiteflies on plant biology, a further trophic level in the analysis was added. In the Mediterranean basin, *M. pygmaeus* spontaneously colonizes tomato crops when pesticide applications are reduced [34,35]. However, as a zoophytophagous insect, it can also feed on the mesophyll of leaves, the tissues of stems, inflorescences, and fruits [36,37]; the suitability of this predator for establishment also varies depending on the species of the host plant and the part of the plant on which the predator lives [38]. Because of this, and bearing in mind the considerable diffusion of this mirid bug in horticultural areas of the Mediterranean basin, we sought to investigate the effects on the morphology and physiology of eggplants through the combined action of *B. tabaci* and *M. pygmaeus*, and we evaluated the modifications to various morphological and physiological parameters of host plants following infestation by *B. tabaci* MED and by a combined presence of this whitefly with its predator *M. pygmaeus*, compared with totally noninfested plants.

## 2. Materials and Methods

### 2.1. Insects and Plants

A colony of *Bemisia tabaci* was collected from an eggplant crop grown under greenhouse conditions in southeast Sicily (Vittoria, province of Ragusa, 36.97134 lat.; 14.424505 lon.). The specimens were then transferred and maintained on eggplant plants in the laboratory under controlled environmental conditions (T = 25 ± 2 °C, RH = 65 ± 5%, and photoperiod of 14L:10D h).

*Macrolophus pygmaeus* individuals came from commercial sources (MIRICAL; Koppert Biological Systems, S.L., Águilas, Murcia, Spain). These were maintained in the laboratory, under the same environmental conditions as those for *B. tabaci*, on eggplants infested by the whitefly. These were also fed every 3 days with eggs of *Ephestia kuehniella* Zeller (Koppert B.V., Berkel en Rodenrijs, BE, The Netherlands).

Host plants (*Solanum melongena* L. cv. Gloria) were grown from seeds germinated and raised in polystyrene planting trays in a nursery. The seedlings were then individually transplanted into black plastic pots (10 cm × 10 cm × 12 cm), using a professional potting soil specific for vegetable sowing, and maintained under controlled environmental conditions in the laboratory (T = 25 ± 2 °C; R.H. = 65 ± 5%, and photoperiod of 14L:10D h) throughout the experiment.

### 2.2. Experimental Design and Sampling

The study was carried out at the laboratories of the Applied Entomology section, Department of Agriculture, Food and Environment (Di3A), University of Catania, Italy, in the period October 2021–January 2022.

The species identity of *B. tabaci* was genetically attained on about 30 whiteflies collected from the rearing described above, before running the test. Using molecular biological methods [39,40], all tested individuals were identified as *B. tabaci* MED, Q2 subclade.

The impact of *B. tabaci* and *M. pygmaeus* on the host plants was assessed on a total of 36 eggplant plants with 6 fully expanded leaves. The trial was set up using a completely randomized design with 12 replicates under each of the following three evaluated conditions (hereafter treatments): noninfested control plants (C); plants infested by *B. tabaci* (PIB); and plants infested by *B. tabaci* where *M. pygmaeus* was also released (PIBM). In order to infest the plants representing the PIB and the PIBM treatments, 4 weeks after transplanting groups of three plants were isolated in netted cages (L × W × H: 60 cm × 60 cm × 60 cm), and 60 unsexed newly emerged (<24 h old) whitefly adults (i.e., 20/plant) collected from the insectary were released on the floor in the center of each cage. The whitefly adults were allowed to lay eggs for 3 days before being removed from the cages by a mouth aspirator (John W. Hock Company, Gainesville, FL, USA).

Next, to assess whether oviposition had occurred, the number of eggs laid was counted on three leaves of each plant, using a stereomicroscope (Olympus Optical Co., Ltd., Tokyo, Japan, SZX-ILLK200). To verify the progress of the infestation, the nymphs fixed on the lower surface of each of the three previously selected leaves were checked 2 weeks after removal of the adults. In line with procedures described in the literature [41,42], 3 weeks after oviposition by *B. tabaci*, 24 unsexed newly emerged *M. pygmaeus* adults (<24 h old) were released on the floor in the center of each cage to test the “pest + zoophytophagous predator” condition (PIBM) (i.e., 8/plant). The mirid adults were allowed to lay eggs for 6 days [43] before being removed from the cages. The monitoring of nymphs’ emergence started 8 days after their release [44] and continued daily for 5 weeks. All newly emerged specimens were also fed with eggs of *E. kuehniella*, glued on a paper strip and provided every 3 days. By following the method described by Sanchez [45], these specimens were counted visually on all entire eggplant plants and were removed from the cages using the mouth aspirator at the end of the experiment (i.e., after about 40 days from the introduction of *M. pygmaeus*). All plants were watered twice a week.

To assess the combined effects of both insects on *S. melongena* development, the height of the plants (PH), the indirect chlorophyll content (ICC), the chlorophyll fluorescence (CF), the dry plant biomass (roots and shoots: RDW and SDW), and the leaf and root areas (LA and RA) were all measured at the end of the experiment. Plant height, expressed in centimeters (cm), was measured from stem base to apex [46] with a ruler. To obtain values for RDW and SDW, expressed in grams, the fresh hypogeal and epigeal biomass was oven-dried (Thermo Fisher Scientific, Langenselbold, Germany, Heratherm OGS100) at 65 °C for 3 days and finally weighed with a high-precision balance (ORMA BC 1000, Orma srl, Milan, Italy; resolution 0.1 g). To calculate the amount of chlorophyll present in the leaf [47]; ICC measurements were taken using a Soil Plant Analysis Development (SPAD-502, Minolta, Sakai, Osaka, Japan) chlorophyll meter on three leaves per plant, which were at principal growth stage 1, according to the BBCH scale [48]. To measure plant stress, the CF data were collected using an OS1-FL Chlorophyll Fluorometer (Opti-Sciences Inc., Hudson, NH, USA). Initially, a leaf in the middle third of each plant was dark-adapted for at least 20 min with detachable leaf clips. Next, the device emitted a saturation pulse through a beam of light, which was read by the device when reflected [49]. The parameter considered in our experiment was F_V_/F_M_, which is the ratio of the variable to the maximum fluorescence after dark-adaptation, which provides information on the functioning of photosystem II (PSII), representing the maximum quantum yield of PSII [50]. In other words, it is a sensitive indicator of plant photosynthetic performance that enables the comparison of plant samples of the same known dark-adapted state using a normalized ratio [51]. The LA and RA of plants were expressed in square centimeters (cm^2^) and determined by ImageJ software (Wayne Rasband—Services Branch, National Institute of Mental Health, Bethesda, MD, USA), which processed the pictures taken by a digital camera (Nikon D850 45.4 megapixel). To monitor the stress state of the plants, ICC and CF were performed monthly for the duration of the test [49].

### 2.3. Data Analysis

Data of parameters relating to the responses of different plants to the three tested conditions were analyzed using a one-way ANOVA. Where significant differences were detected, the means were separated by Tukey’s HSD test (*p* < 0.05). Statistical analysis was carried out using the program Statistica (StatSoft, TIBCO Software Inc., Tulsa, OK, USA).

## 3. Results

**Figure 1:**
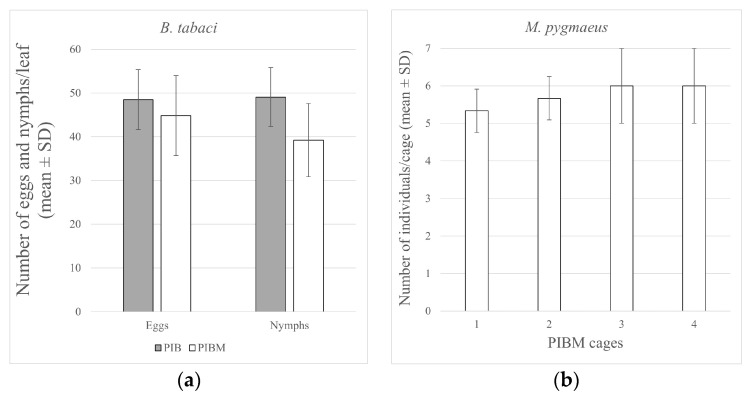
Mean number (±SE) of (**a**) *B. tabaci* eggs laid and nymphs fixed on the lower surface of each of the three selected leaves per plant under both PIB and PIBM conditions and (**b**) *M. pygmaeus* specimens per PIBM cage.

The mean number of eggs laid by the whitefly adults on the lower surface of each of the three selected leaves per plant after 3 days of exposition was 46.6 ± 6.94 (average: 2.2 eggs/cm^2^). Further, 17 days after the adults were released, the average number of nymphs was 44.1 ± 9.98 (mean: 2.1 nymphs/cm^2^). These results confirmed that oviposition and the progress of infestation evenly occurred in all plants under both PIB and PIBM conditions (Figure 1a).

The average total density of *M. pygmaeus*, expressed as the number of specimens per plant 40 days after release, was 5.7 ± 0.7 insects/plant (Figure 1b). About 2 weeks after the release of *M. pygmaeus*, its first-instar nymphs appeared, and these became adults by the end of the experiment.

**Figure 2:**
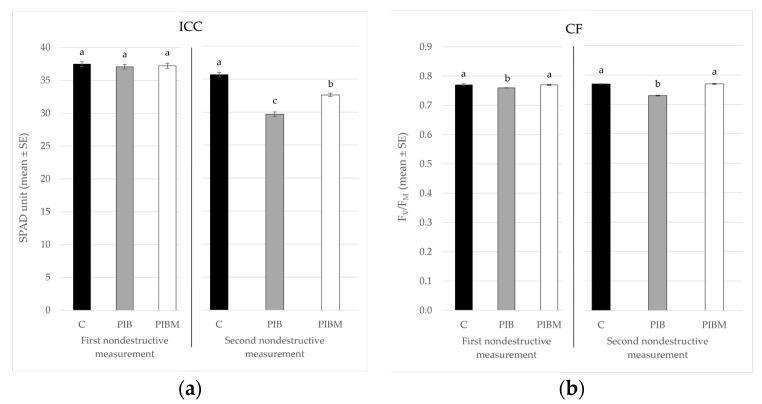
Mean values (±SE) of (**a**) the indirect chlorophyll content (ICC) and (**b**) the chlorophyll fluorescence (CF) detected in the first and second nondestructive measurements performed on noninfested control plants (C), eggplants infested by *B. tabaci* (PIB), and host plants in the presence of both *B. tabaci* and *M. pygmaeus* (PIBM). Different letters indicate statistically significant differences at *p* < 0.05.

The stress state of plants during the test was indicated by the first nondestructive measurement taken 1 month after the beginning of the experiment. This showed no statistical differences in the ICC values (Table 1) between C, PIB, and PIBM modalities (*F*_2,33_ = 0.32; *p* < 0.727) (Figure 2a). In contrast, CF values recorded in the same period (Table 1) showed significant differences between PIB and the other two conditions, C and PIBM (*F*_2,33_ = 4.89; *p* < 0.0138) (Figure 2b). Starting from the second measurement carried out 2 months after the beginning of the test, the ICC parameter was influenced by the presence of the two insects (Table 1), revealing statistical differences among the three conditions (*F*_2,33_ = 86.31; *p* < 0.001) (Figure 2a). However, with respect to the CF parameter (Table 1), in the second nondestructive measurement, the statistical differences found in the first measurement were maintained (*F*_2,33_ = 91.9; *p* < 0.001), with the lowest mean values recorded in plants infested by *B. tabaci* (Figure 2b).

**Figure 3:**
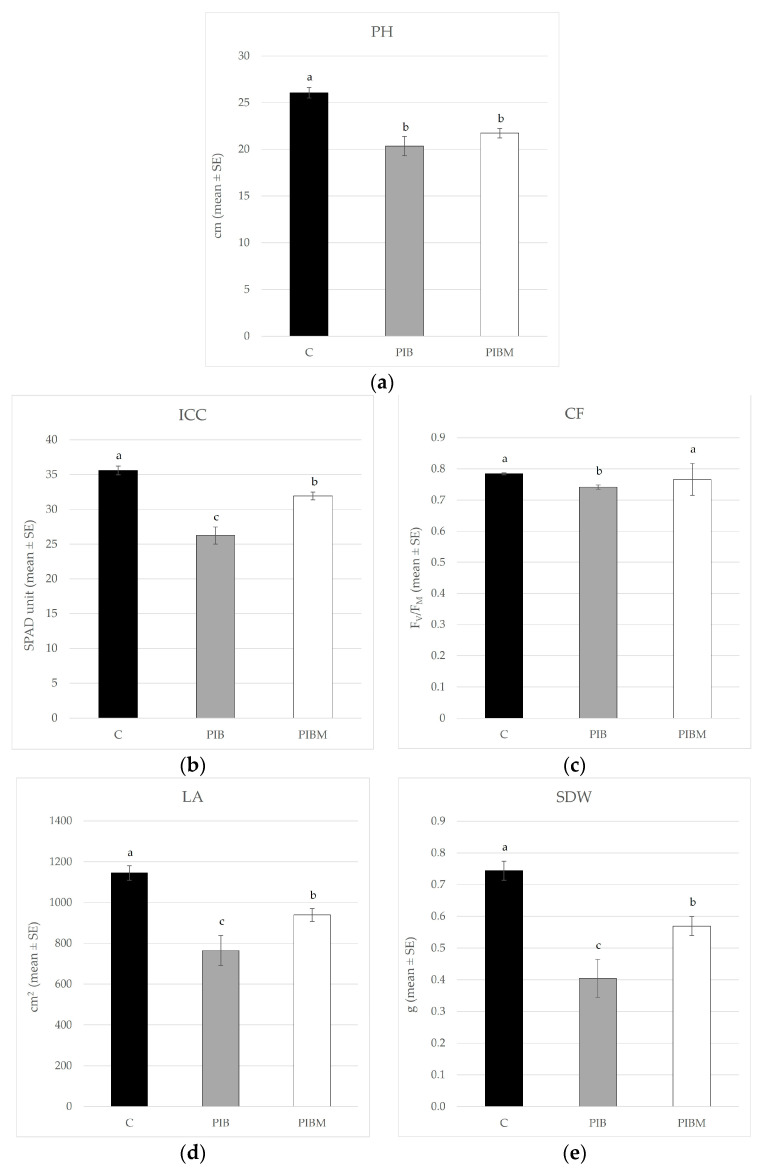

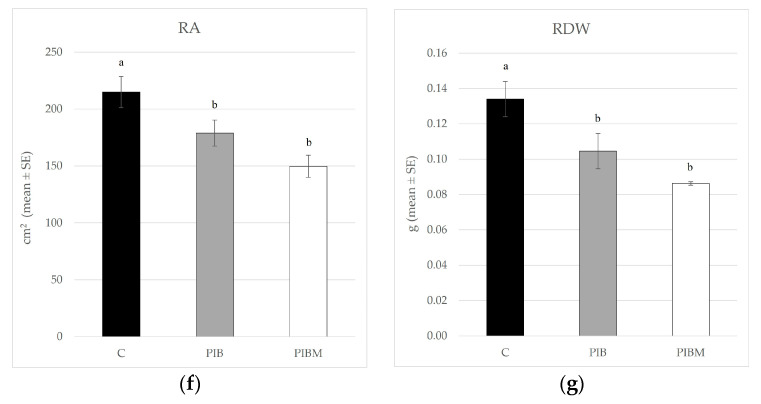
Mean values (±SE) of (**a**) plant height (PH), (**b**) indirect chlorophyll content (ICC), (**c**) chlorophyll fluorescence (CF), (**d**) leaf area (LA), (**e**) shoot dry weight (SDW), (**f**) root area (RA), and (**g**) root dry weight (RDW) of noninfested control plants (C), host plants infested by *B. tabaci* (PIB), and eggplants in the presence of both whitefly and *M. pygmaeus* (PIBM). Different letters indicate statistically significant differences at *p* < 0.01.

At the end of the experiment, the values for all considered plant physiology and morphology parameters showed statistical differences between the conditions examined, as reported in Table 2. Overall, plant height (PH) was negatively affected (*F*_2,33_ = 16.297; *p* < 0.001) by the presence of both the insect species, with higher mean values recorded in the noninfested plants (Figure 3a).

The release of *B. tabaci* (PIB) caused a clear detrimental effect on indirect chlorophyll content (ICC) in the leaves of infested plants, which was statistically different when compared with the other two tested modalities (*F*_2,33_ = 29.728; *p* < 0.001); indeed, in the presence of the predators (PIBM), the plants exhibited a slightly higher chlorophyll content, though it was still less than that of the noninfested plants (C) (Figure 3b).

Similarly, the CF values indicating the plant’s photosynthetic performances followed a broadly similar trend; in this case, a data analysis revealed a statistically significant decrease in the CF values (*F*_2,33_ = 16.159; *p* < 0.001) of the PIB treatment in comparison with PIBM treatment (i.e., when the predator was released in the cage) that was not statistically different from the noninfested plants of the C condition (Figure 3c).

Even in the case of the leaf area (LA) of plants, there was a statistical difference between the three tested conditions (*F*_2,33_ = 13.45; *p* < 0.001); in particular, the lowest mean values of the PIB condition suggested that the presence of the predator limited the damage caused by the whitefly (Figure 3d).

Similarly, in the case of the SDW parameter, the analysis revealed a statistical difference among plants under the three conditions (*F*_2,33_ = 17.12; *p* < 0.001) (Figure 3e), where those of the PIB condition, in the absence of *M. pygmaeus*, once again exhibited the lowest values.

As was the case with plant height, the root area and root dry weight (i.e., RA and RDW) were also negatively influenced by the presence of both insect species. In particular, the highest mean values were recorded in the noninfested plants, with statistically significant differences in comparison with the plants of the other two treatments (*F*_2,33_ = 7.74; *p* < 0.0017 and *F*_2,33_ = 6.42; *p* < 0.0044—for RA and RDW, respectively) (Figure 3f,g).

## 4. Discussion

Integrated pest management aims to guide agriculture strategies by controlling arthropod infestations through the optimal selection of host plants [52] and management of the activity of natural enemies [53]. In this context, the responses of host plants to the presence of pests have often been studied, but it remains unclear how plants respond to the zoophytophagy of predatory omnivorous insects [54].

The present research indicates that the mirid predator *M. pygmaeus* exerts a significant influence on some physiological and morphological traits of *S. melongena* (e.g., indirect chlorophyll content, chlorophyll fluorescence, root area and dry weight, etc.) that have been poorly investigated so far.

It is well known that infestation by *B. tabaci* affects the quantity and quality of yields in many varieties of vegetable crops [55]. The means by which whitefly infestations exert a negative effect on horticultural species (eggplant and tomato) have also been confirmed in a recent study [33]. The findings reported here show that the presence of *M. pygmaeus* reduces the negative effects of the whitefly on plants [38,46], resulting in significantly higher values of indirect chlorophyll content, chlorophyll fluorescence, leaf area, and shoot dry weight in the PIBM condition compared with the PIB. In this regard, according to Pappas [54], zoophytophagous hemipterans, such as *Orius insidiosus* (Say) and pentatomids, feed on plants mainly to acquire water from the xylem and also potentially to obtain nutrients from the mesophyll or pollen, most likely causing only some minor cell wounding on leaves. Feeding by mirid bugs therefore depends on the plant sap and not only on prey. This explains the greater suitability of the eggplant compared with other vegetable crops, which results in a relatively longer survival of the predator even in the absence of prey [55]. However, it is also known that plant sap may influence the taste of prey individuals, making them either more or less attractive and desirable to the predators, so that eggplants might generate better prey than other vegetable crops do [56]. Furthermore, the presence of the eggs of *E. kuehniella*, another optimal food source for *M. pygmaeus*, may have helped the predator to successfully establish and increase a stable population [57].

However, as observed by Bresch [46] in a study of tomato and tobacco plants infested by *Trialeurodes vaporariuorum* (Westwood), the mirid predator *M. pygmaeus* cannot always significantly reduce the negative impact of the pest. Indeed, the results of the present study reveal that *M. pygmaeus* was unable to limit the effect of *B. tabaci* on PH and on the characteristics of the roots (i.e., RA and RDW) that were not significantly different from those obtained in the PIB condition.

Insect pests can trigger the production of deterrents or toxic secondary metabolites that reduce the suitability of plant tissues for further insect colonization and may compromise the ability of the plant to activate certain resistance-related pathways [58,59,60]. This could be the case with *B. tabaci*, which induces the activation of salicylic acid (SA) [61,62], a phytohormone able to suppress the activation of jasmonic acid (JA) [53,63]. However, the latter phytohormone can be induced by mirid insect activities (e.g., oviposition by adult females and feeding by *M. pygmaeus* nymphs) [54] and functions in the mediation of plant responses. Specifically, the root stimulation of JA responses, following shoot damage, is completely dependent on hormone translocation [64]; thus, the phloem transport system is crucial to allocate resources among plant tissues and organs and move the jasmonates, which accumulate in vascular bundles after wounding [65,66,67].

Our findings may be seen in line with the results obtained by Schulze [64], who reported that even if, following wounding, shoot-produced jasmonates on *Arabidopsis thaliana* (L.) Heynh. move downward into the root through the phloem, the wounding on shoots is not always able to trigger the expression of JA marker genes in roots of all genotypes. Because JA may produce a local and nonsystemic effect [68] and because our eggplants (PIBM) were exposed to *M. pygmaeus* for mating and oviposition for only 3 days, it is probable that compounds related to plant defense were not transferred up to the roots during the experimental period. As indicated by Zhang [68], a longer exposure of host plants to mirids, or exposure to a greater density, could result in more-evident differences in phytohormone concentrations in various parts of plants, with consequent increases in root area and root dry weight, as was the case in our roots.

Although our results offer a preliminary insight into multitrophic interactions and mechanisms among host plant, pest, and predator, the specific response of whole eggplants to the presence of *M. pygmaeus* remains unclear. The wound-induced responses of plants are often modified by the perception of herbivore-specific elicitors, and this may be the case for zoophytophagous omnivores, especially within the Miridae, as they produce many different salivary enzymes [69,70]. Further investigations are needed to more deeply study how phytophagy by *M. pygmaeus* directly and indirectly affects host plants and whether *B. tabaci* is able to suppress or resist plant defenses stimulated by the predator.

## Figures and Tables

**Table 1:** Stress state of plants recorded during the first and second nondestructive measurements, as indicated by the calculation of the amount of chlorophyll present in the leaf (ICC) and by calculation of the plant’s photosynthetic performance (CF).

Treatment	1st ICC (SPAD Unit ± SE)	2nd ICC (SPAD Unit ± SE)	1st CF (F_V_/F_M_ ± SE)	2nd CF (F_V_/F_M_ ± SE)
C	37.48 ± 0.36 a	35.68 ± 0.34 a	0.77 ± 0.004 a	0.77 ± 0.001 a
PIB	37.07 ± 0.33 a	29.68 ± 0.35 c	0.76 ± 0.001 b	0.73 ± 0.002 b
PIBM	37.23 ± 0.39 a	32.61 ± 0.27 b	0.77 ± 0.002 a	0.77 ± 0.002 a
*F*; df; *p*	0.32; 2, 33; <0.727	86.31; 2, 33; <0.001	4.89; 2, 33; <0.0138	91.9; 2, 33; <0.001

ANOVA parameters are reported for each test condition. Within each column, data followed by different letters are significantly different (*p* < 0.05, Tukey’s HSD test).

**Table 2:** Impact of the biological activity of *Bemisia tabaci* (PIB), either alone or in association with *Macrolophus pygmaeus* (PIBM), on the main morphological and physiological parameters of eggplants. (PH—plant height; ICC—indirect chlorophyll content; CF—chlorophyll fluorescence; LA—leaf area; RA—root area; SDW—dry shoot biomass; RDW—dry root biomass).

Treatment	PH (cm ± SE)	ICC (SPAD Unit ± SE)	CF (F_V_/F_M_ ± SE)	LA (cm^2^ ± SE)	SDW (g ± SE)	RA (cm^2^ ± SE)	RDW (g ± SE)
C	26.07 ± 0.57 a	35.60 ± 0.64 a	0.78 ± 0.003 a	1141.49 ± 35.67 a	0.74 ± 0.03 a	214.90 ± 13.69 a	0.13 ± 0.01 a
PIB	20.35 ± 1.03 b	26.23 ± 1.23 c	0.74 ± 0.007 b	765.17 ± 74.21 c	0.40 ± 0.06 c	178.91 ± 11.38 b	0.11 ± 0.01 b
PIBM	21.73± 0.51 b	31.91 ± 0.56 b	0.77 ± 0.051 a	939.17 ± 31.81 b	0.57 ± 0.03 b	149.83 ± 9.73 b	0.08 ± 0.001 b
*F*; df; *p*	16.297; 2, 33; <0.001	29.728; 2, 33; <0.001	16.159; 2, 33; <0.001	13.45; 2, 33; <0.001	17.12; 2, 33; <0.001	7.74; 2, 33; <0.0017	6.42; 2, 33; <0.0044

ANOVA parameters are reported for each test condition. Within each column, data followed by different letters are significantly different (*p* < 0.05, Tukey’s HSD test).

## Data Availability

The data presented in this study are available on request from the corresponding author.
